# Identifying mechanisms of regulation to model carbon flux during heat stress and generate testable hypotheses

**DOI:** 10.1371/journal.pone.0205824

**Published:** 2018-10-26

**Authors:** Allen H. Hubbard, Xiaoke Zhang, Sara Jastrebski, Susan J. Lamont, Abhyudai Singh, Carl J. Schmidt

**Affiliations:** 1 Donald Danforth Plant Science Center, Saint Louis, Missouri, United States of America; 2 Department of Statistics, George Washington University, Washington, District of Columbia, Unites States of America; 3 Department of Animal and Food Sciences, University of Delaware, Newark, Delaware, United States of America; 4 Department of Animal Science, Iowa State University, Ames, Iowa, United States of America; 5 Department of Electrical Engineering and Computer Science, University of Delaware, Newark, Delaware, United States of America; University of Illinois, UNITED STATES

## Abstract

Understanding biological response to stimuli requires identifying mechanisms that coordinate changes across pathways. One of the promises of multi-omics studies is achieving this level of insight by simultaneously identifying different levels of regulation. However, computational approaches to integrate multiple types of data are lacking. An effective systems biology approach would be one that uses statistical methods to detect signatures of relevant network motifs and then builds metabolic circuits from these components to model shifting regulatory dynamics. For example, transcriptome and metabolome data complement one another in terms of their ability to describe shifts in physiology. Here, we extend a previously described linear-modeling based method used to identify single nucleotide polymorphisms (SNPs) associated with metabolic changes. We apply this strategy to link changes in sulfur, amino acid and lipid production under heat stress by relating ratios of compounds to potential precursors and regulators. This approach provides integration of multi-omics data to link previously described, discrete units of regulation into functional pathways and identifies novel biology relevant to the heat stress response, in addition to generating hypotheses.

## Introduction

The highly conserved heat stress response has been extensively studied in organisms across taxa. Understanding how to mitigate effects of hyperthermia has important applications in a variety of disciplines. For example, heat stroke is a common, severe complication of acute hyperthermia and a stronger biological understanding of its underpinnings could lead to more effective therapies [1]. In the agricultural setting, prolonged heat stress—such as that encountered by livestock during heat waves—can decrease feed efficiency and animal growth and cause significant commercial losses in meat production [2]. Additionally, heat stress has been known to exert systemic physiological consequences such as changes in egg production and immune cell counts [3]. While many of physiological consequences of hyperthermia are understood, a strong mechanistic description, particularly in the commercially important broiler chicken, the subject of this study, is lacking.

Metabolic shifts during heat stress involve a number of signaling cascades [4]. These include the unfolded protein response and both pro and anti-apoptotic pathways [5]. Increasing evidence suggests that biologically active lipids play an important function during heat stress, as signaling agents and maintaining cell membrane integrity [6]. Establishing the relationship between lipid metabolism and other well-characterized heat responsive pathways would improve understanding of the flow of resources to different types of metabolites. This could provide a model describing how small carbon precursors are selectively routed to various fates necessary to sustain signaling, energy production, and other processes that must undergo dynamic shifts under heat stress.

Viewing carbon flow as a circuit with dynamics that are affected by gene expression changes produces an effective description and identifies testable biological hypotheses. This perspective describes the mechanisms that manage and create resource pools in the form of biochemically valuable carbon backbones. This includes cysteine and other catabolized amino acids, that are selectively incorporated into various biologically active of molecules. The model that we construct describes the interconnection between production of antioxidants, increased sugar metabolism, and production of signaling and structural lipids.

Redirection of carbon backbones occurs at specific points of regulation where molecules are processed into one of two or more available metabolic fates. This regulation can be described by linear models. We have previously introduced this type of regulation in the form of metabolic forks, but have now extended them to build pathway level models [7]. Importantly, this arrangement detects how gene expression patterns implement regulatory logic by selectively directing resources.

## Materials and methods

We first subsetted metabolite measurements for compounds representing sulfur, lipid, and sugar metabolism because these are major processes influenced by heat stress [8]. This initial step of feature selection reduced metabolite data from 600 compounds to approximately 60 to serve as candidates in linear models. Our analysis deliberately focused on regulatory circuits controlling metabolism involving only the selected components of tissue enriched genes and metabolites linking sulfur and lipid metabolism. Details regarding bird experimental treatments and library preparation from liver tissue can be found in the Supplementary Information. The samples described in the study are from broiler liver tissue, as this is an important location in the bird for regulation of processes affected by heat stress [8].

### Iterative linear models and metabolic forks

**Fig 1:**
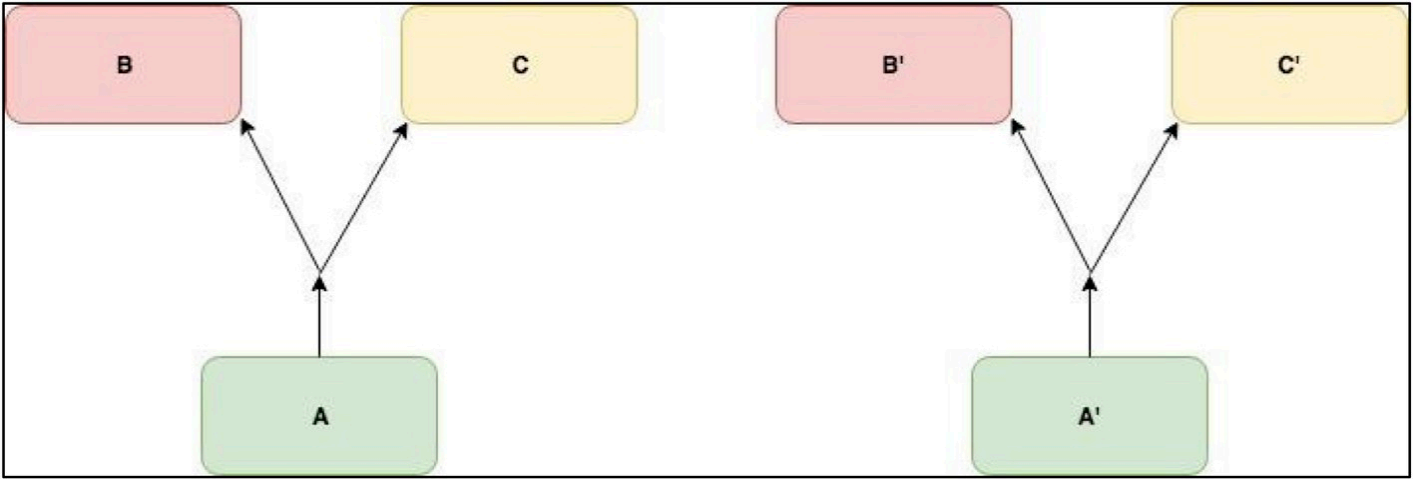
An example of two potential metabolic forks detected from the data through linear models. Each one represents a triplet whose models $A∼\left(\frac{B}{C}\right)$ and $\left(\frac{B}{C}\right)∼A$ have significant interaction terms between control and heat stress conditions.

We evaluated levels of compounds representing sulfur, lipid, and sugar metabolism as these are major processes influenced by heat stress, in order to determine metabolic forks among these systems and clarify how these types of metabolism relate to one another. Values for a correlation function of the form $cor\;\left(A,(\frac{B}{C})\right)$ were calculated under control and heat stress conditions, with A, B and C representing the levels of metabolites or gene transcripts (Fig 1). This method demonstrates that the correlation between compound A and the ratio $\frac{B}{C}$ can differ between control and experimental conditions when there is heat responsive regulation of members of the triplet, as the members of many of the selected triplets were significantly impacted by heat stress. The most biologically informative triplets of the form A, B, and C often represent sets of precursors and their resulting metabolic products. Precursor-product pairs were frequently found in triplets. These include, for example, glutathione and its known precursor cysteine [9] as well as other triplets involving taurine which shares also depends on cysteine as a precursor [10]. Because many compounds in separate triplets share precursors, it is possible to use resulting linear models to detect differential routing of resources.

**Fig 2:**
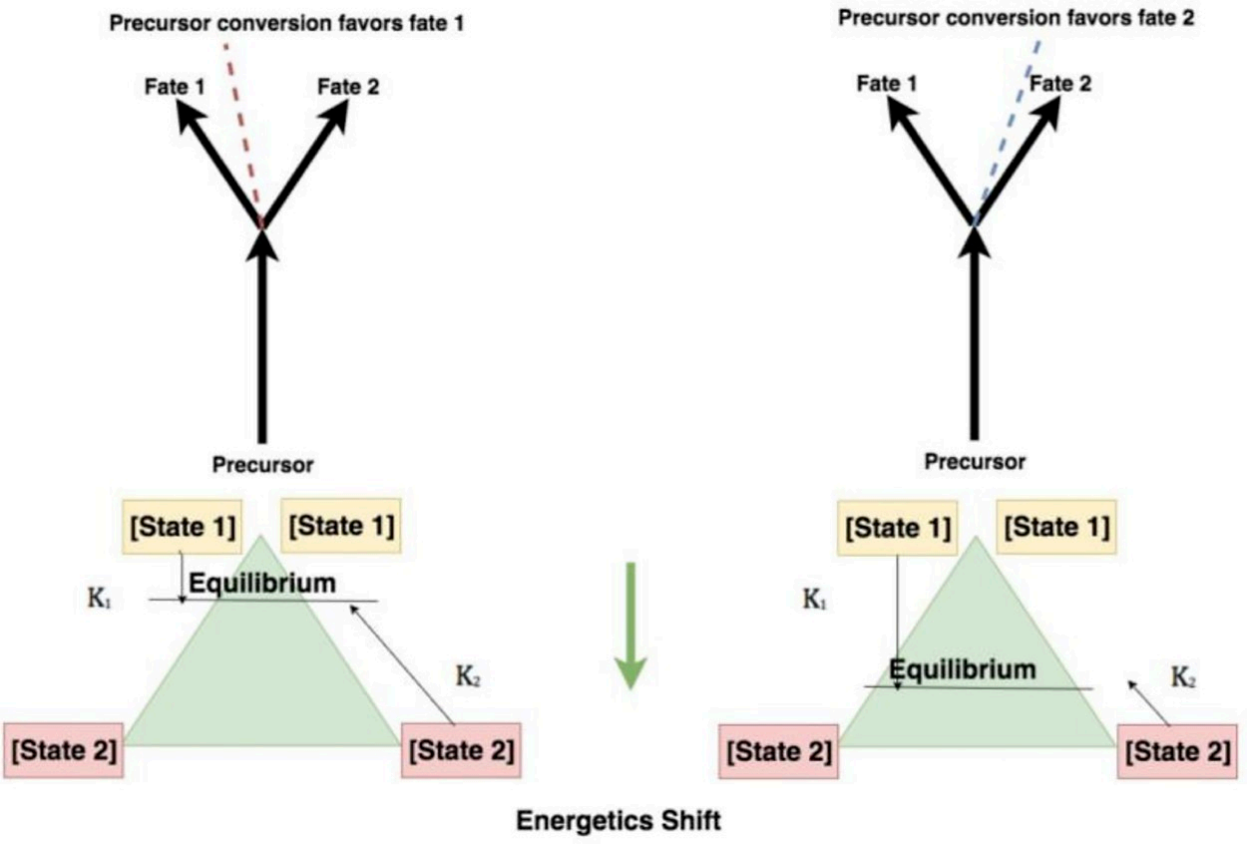
Metabolic forks, representing different favorabilities for the metabolic fates at regulatory branch points. A shift in regulation changes the energetic favorability in favor of one route of the metabolic fork.

Ratios of compounds were used in these functions and subsequent models because they are more sensitive to detecting points of potential regulation for diverging metabolic routes [11]. A biochemical interpretation of these functions is provided in Fig 2. Triplets whose difference in value for the correlation function was 1.2 or greater between control and experimental conditions, i.e. $\left|cor\;(A,(\frac{B}{C}))\;heat-cor\;(A,(\frac{B}{C}))\;control\right|>1.2$, were selected as representing possible metabolic forks. A threshold was chosen because of the trade-off between identifying a diverse set of triplets and the need to achieve stringency such that resulting linear models of the triplet were likely to have significant interaction terms. Linear models were then used to detect differential behavior under heat stress and to identify triplets with significant interaction terms–indicating different slopes between control and heat stress conditions.

**Fig 3:**
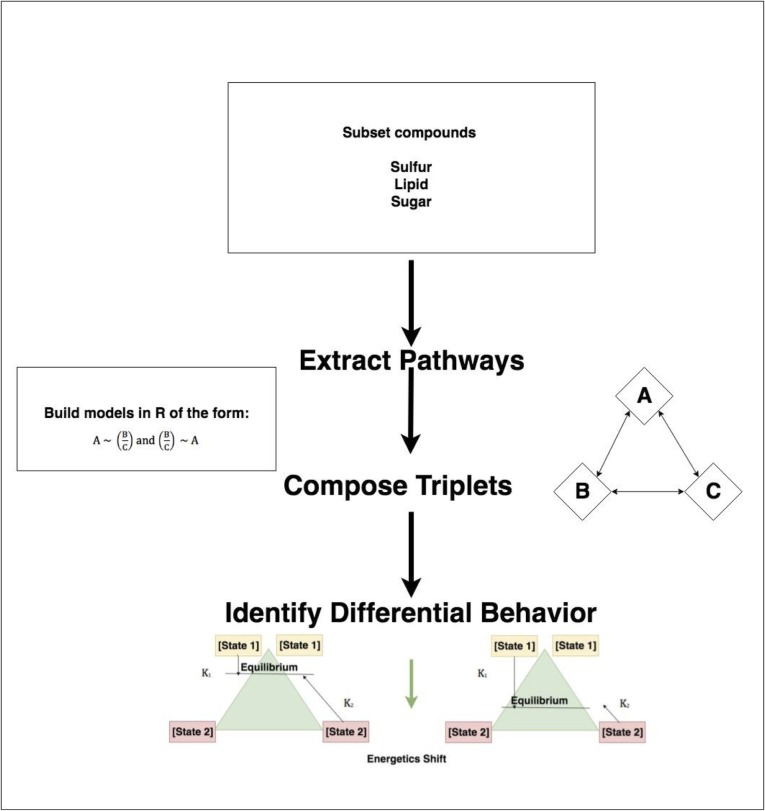
Workflow to identify triplets of compounds that regulate sulfur and lipid metabolism.

**Fig 4:**
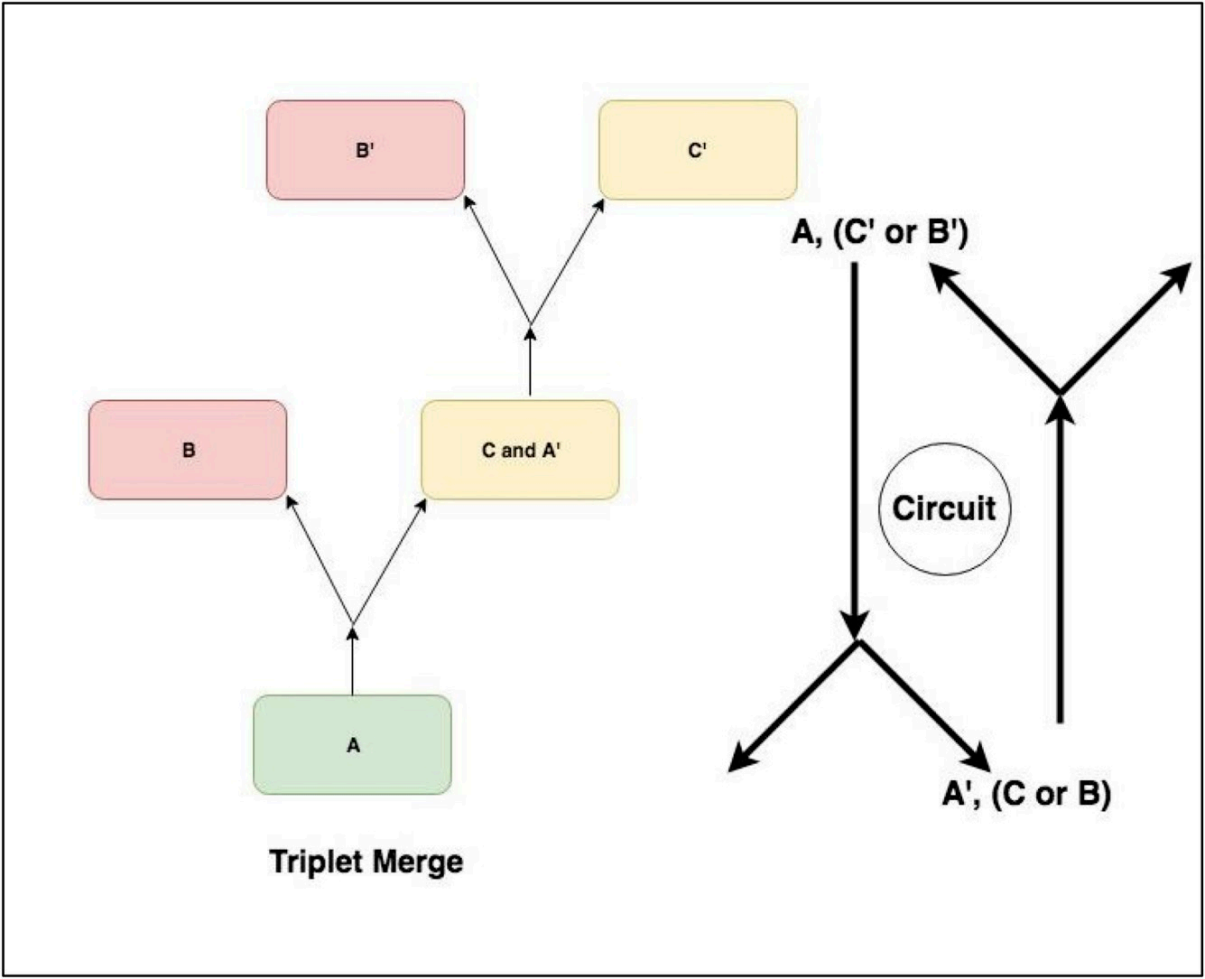
Metabolic forks being merged. Metabolic forks are joined into potential pathways by identifying forks that share overlapping members of triplets.

The threshold of 1.2 for identifying potentially interesting triplets was ad-hoc, with the linear models being used to determine a p-value for the differential regulation between control and experimental conditions. Thus, ad-hoc correlation functions were used merely as screening to propose candidates for more rigorous evaluation by linear models. To be considered as a pathway element and incorporated into a circuit, the interaction term must be significant for both models of the form $A∼\left(\frac{B}{C}\right)$ and $\left(\frac{B}{C}\right)∼A$. This stringent heuristic is chosen because of ambiguity regarding directionality of the relationship between $\left(\frac{B}{C}\right)$ and A. For example, while ratios are more sensitive to detecting relationships between possible sets of precursors and a product, it is not always clear which relationships among the triplet are causal and which are correlative. While the reliance on linear models does enforce an assumption of linearity, such an assumption is consistent with the use of correlation, which also measures linear relationships, to identify differential regulation of triplets (Fig 3). After the formulation of linear models, triplets were merged (Fig 4) with one another to generate pathways. We focus on three involving sulfur and lipid regulation, because regulation associated with these triplets represents the functioning pathway of lipid and antioxidant regulation also described by complementary transcriptome data. Regarding components of a metabolic fork, in terms of their relationship to one another as precursors and products, these hypotheses are necessarily associative and not always causal. However, confidence in the proposed directionality of relationships can be strengthened by gene expression changes. Per existing methods, all data was log transformed before modeling [12]. Once libraries were sequenced, data were processed using an in-house pipeline and fragments per kilobase per million mapped reads (FPKM) values were determined. Differential expression was determined by using the standard t.test function in R.

### From merged triplets to pathways

By merging together forks it is possible to identify small, functional units that may be critical elements of pathways. We demonstrate that integrating these isolated units to form controlled regulatory systems can identify circuits of carbon and sulfur regulation. Importantly, linear models relying on the ratios of metabolites identify differential behavior not detectable using raw expression measurements alone. This may be due to reductions in variance [1] as well as an ability to capture underlying biology by being more sensitive to fluxes down each metabolic pathway. Importantly, these models can then be joined to create a larger circuit of regulation. In these types of models, elements involved in each metabolic fork also play a role in the functioning of other metabolic forks. The biochemical interpretation of each metabolic fork, and the joining of multiple examples to form circuits captures the intuition and biochemistry of pathways.

## Results

**Fig 5:**
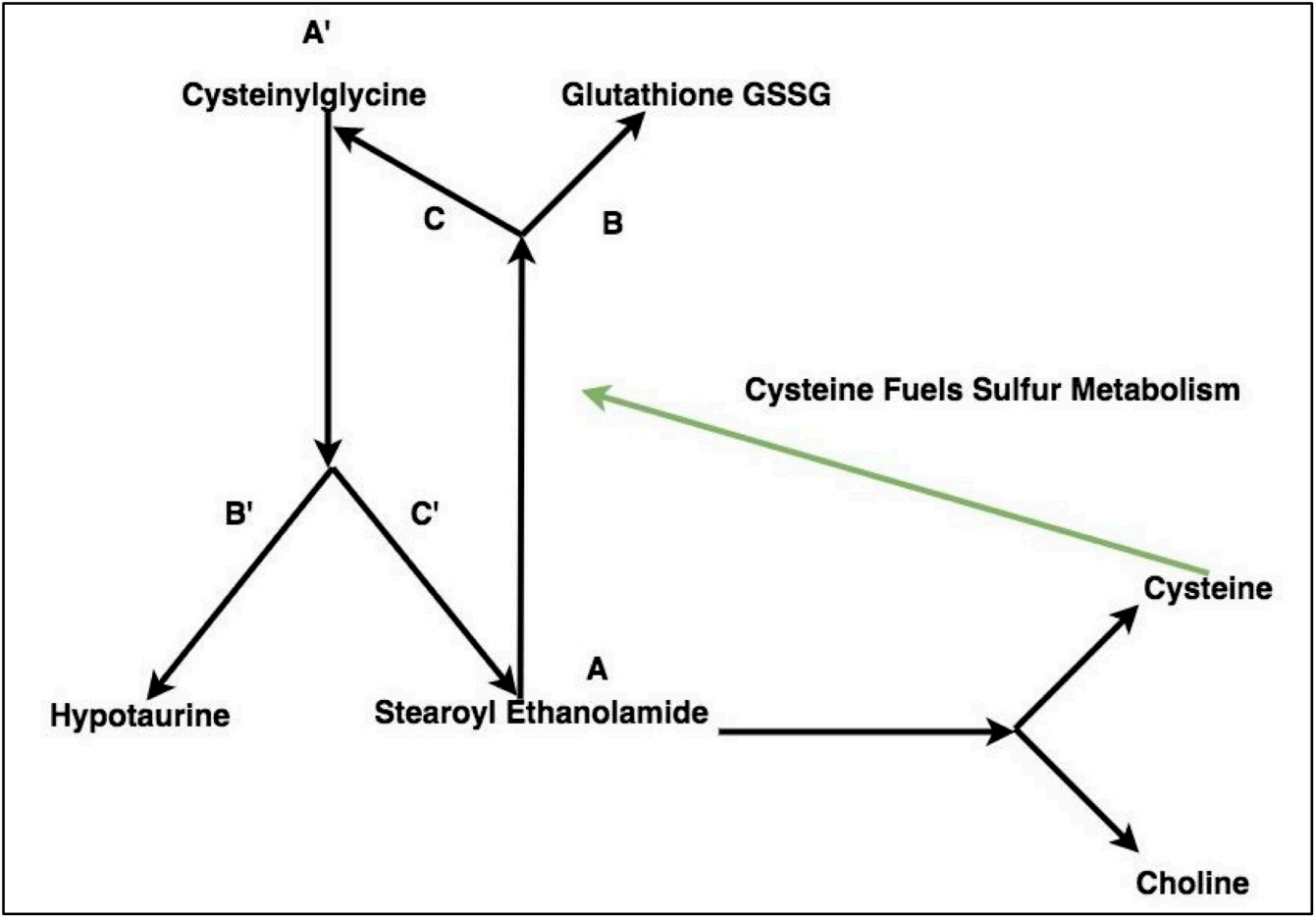
Extended circuit based off of merging of triplets.

A full mechanism relating sulfur, lipid, and antioxidant activities to one another can be constructed by linking several triplets (Fig 5).

**Fig 6:**
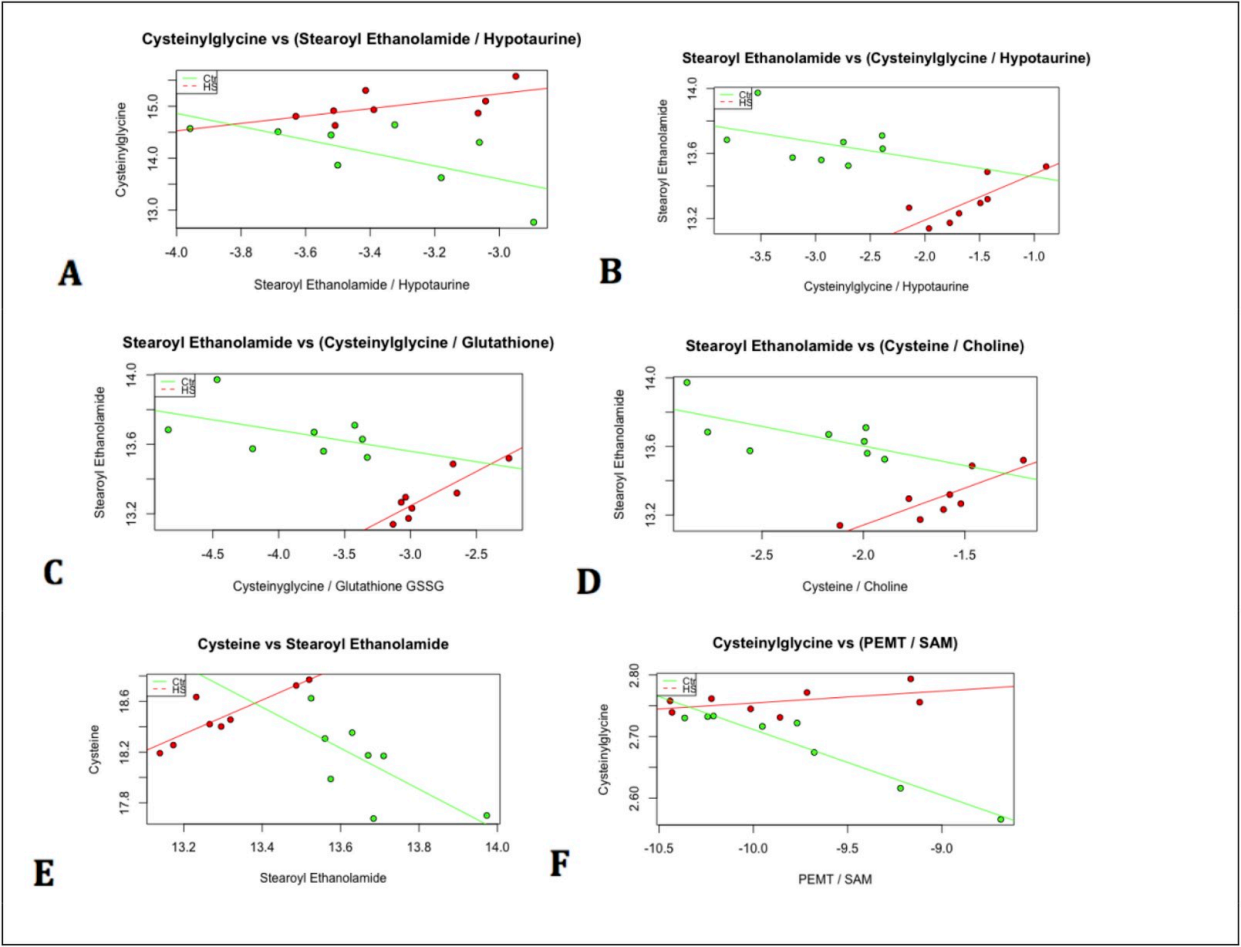
A-F. Metabolic forks and related models. Linear models detect differential behavior of the metabolic forks that comprise the circuit. Figs (6–9) discuss each branch-point in detail. All p-values for relevant interaction terms are less than .05. Data points that are green represent measurements from control conditions, whereas red data points represent measurements from heat stressed birds.

**Fig 7:**
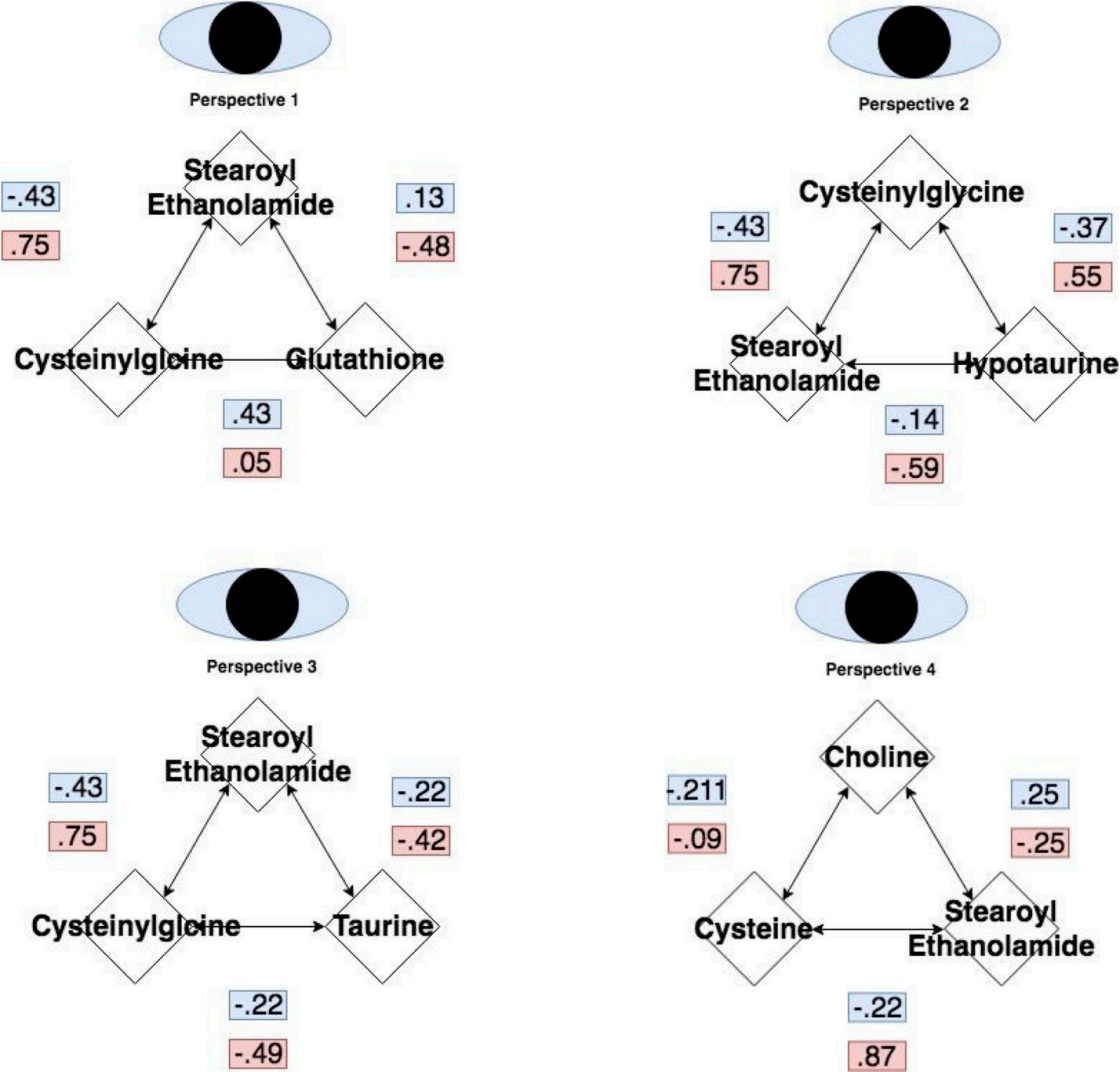
Elements of all metabolic forks that have been joined together describe a pathway that regulated by changes in gene expression. Pairwise correlations between all members of each triplet under heat stress (red) and control conditions (blue) are shown.

This is done by joining triplets (Fig 5) that share at least one overlapping element and whose linear models exhibit differential behavior under heat stress (p-value for interaction term must be < .05). This organizes sets of linear models (Figs 6 and 7) into a more comprehensive pathway representation. The resulting circuit describes, de-novo from the data, relationships between lipid and anti-oxidant compounds. These predictions are consistent with previous research relating hypercysteinemia and hyperlipidemia to one another [13]. However, these relationships have not been previously established as components of the heat stress response, and are thus novel contributions to the best of our knowledge.

Specifically, we detect relationships between stearoyl ethanolamide and the known precursor-product pairs cysteinylglycine and glutathione [10]. This coupling between the lipid stearoyl ethanolamide and the sulfur metabolites cysteinylglcine and gluthatione changes under heat stress conditions (p-value = .00523 for the interaction term in the model stearoyl ethanolamide as a function of the ratio $\frac{cysteinylglycine}{glutatione}$ and p-value = .0251 for the interaction term in the model $\frac{cysteinylglycine}{glutatione}$ as a function of stearoyl ethanolamide). This relationship, which describes selective utilization of cysteine for gluthathione related antioxidant processes, is consistent with linear models for other metabolic forks involving sulfur containing metabolites such as hypotaurine (p-value = .0176 for the interaction term in the model stearoyl ethanolamide as a function of the ratio $\frac{cysteinylglycine}{hypotaurine}$ and p-value = .0349 for the interaction term in the model $\frac{cysteinylglycine}{hypotaurine}$ as a function of stearoyl ethanolamide).

In these models (described in detail in Figs 6 and 7), cysteine levels which are increased under heat stress (p-value = .0227) encourage sulfur metabolism which is also coupled to specific lipid and antioxidant production via changes in expression of key regulatory genes. For example, changes related to cysteine metabolism occur concurrently with decreased levels of choline-derived signaling and structural lipids, as well as changes in gene expression for enzymes related to these processes. Choline oxidase (ChO), which directs choline to sulfur metabolism [14] is upregulated under heat stress (p-value = .0167). Choline kinase (ChoK), which directs choline to production of lipids such as phosphatidylcholine and signaling lipids such as sphingomyelins [15] is downregulated under heat stress (p-value = .0184). Concurrently, classes of sphingomyelin derived lipids are also found at lower levels under heat stress. These include sphingomyelin species such as sphingomyelin (d18:1/21:0, d17:1/22:0, d16:1/23:0) (p-value = 0.000410491), sphingomyelin (d18:1/22:1, d18:2/22:0, d16:1/24:1) (p-value = .001389259), sphingomyelin (d18:1/24:1, d18:2/24:0) (p-value = 9.63E-05) and many other choline derived species (S1 Table). Choline levels themselves are lower under heat stress (p-value = 0.00108).

Differential behavior at each of the forks associated with changes in downstream lipids such as sphingomyelins can be seen clearly in the series of linear models, each of which demonstrate significant interaction terms changing patterns of pairwise correlations under heat stress (Figs 6A–6F and 7). These branch points can be placed in context of known biology to generate a regulatory model that incorporates both transcriptome and metabolome measurements. Genes that regulate the metabolism of each metabolite in the network skeleton can be located on the skeleton in order to flesh out a full pathway model.

## Discussion

Linear models involving metabolites and pairs of precursor-products that demonstrate significant interaction terms can be related to each other through overlapping components. The resulting collection of linear models describes shifts in lipid, cysteine and glutathione production. When combined with transcriptome data, a metabolic circuit emerges that effectively exploits both transcriptome and metabolome data. While metabolite data is mostly used to construct linear models, transcriptome data identifies gene expression changes consistent with the behavior of these models.

**Fig 8:**
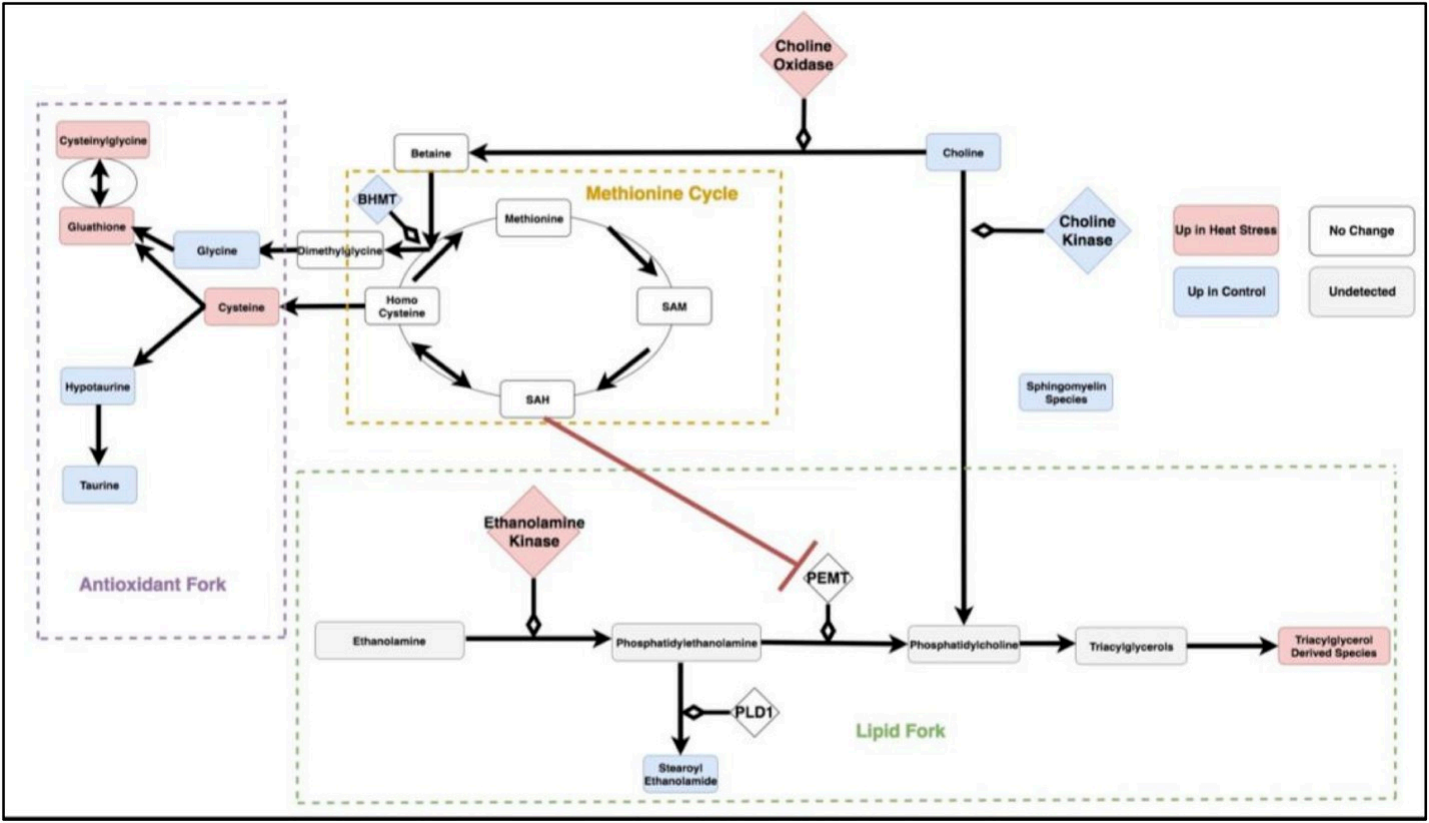
The circuit components as modules summarized by the three categories of antioxidant, lipid and methionine metabolism. SAM: S-Adenosyl-L-methionine, SAH: S-Adenosyl-L-homocysteine,Glutathione GSSG: Glutathione Disulfide, PEMT: Phosphatidylethanolamine N-methyltransferase, BHMT: Betaine—Homocysteine S-Methyltransferase, PLD1: Phospholipase D-1, PEMT: Phosphatidylethanolamine N-methyltransferase.

Fig 8 is a metabolic circuit relating lipid, cysteine and glutathione production that results by considering the behavior of each linear model (depicted individually in Fig 6A–6F), as well as differential expression of important genes regulating components of the linear models. Many of these models describe the incorporation of sulfur into biologically important molecules in a way that is coupled with lipid metabolism. For example, cysteine demonstrates a strong correlation with the lipid molecule stearoyl ethanolamide (.87) under heat stress (Fig 7). Cysteine, which fuels many sulfur processing pathways such as glutathione production, is the only amino acid increased under chronic heat stress (p-value is .0227). Stearoyl ethanolamide levels, however, are lower under heat stress (.000499). The circuit in Fig 8, incorporatin joined triplets as well as gene expression changes, is complex enough to describe several mechanisms by which pools of cysteine can be coupled to the remaining production of lipids attenuated by heat stress. These are reflected in the behavior of the linear models for relevant triplets.

For example, evidence for the fine-tuning of sulfur homeostasis with lipid levels is indicated by the significant interaction terms in the linear model relating the antioxidant cysteinylglycine and the ratio of expression of the ethanolamine processing gene PEMT and sulfur derived metabolite SAM (p-value = .000502 for the interaction term of the model cysteinylglycine as a function of the ratio $\frac{PEMT\;Expression}{SAM}$ and p-value = .0139 for the interaction term of the model $\frac{PEMT\;Expression}{SAM}$ as a function of cysteinylglycine). SAM is a metabolite leveraged for transmethylation associated with the methionine cycle (Fig 8), transsulfination and amino propylation [16]. Metabolism of SAM into SAH has been previously linked to inhibition of PEMT [13]. Thus, increase in the antioxidant cysteinylglycine correlated with the ratio of PEMT/SAM is consistent with coupling of sulfur metabolism and lipid molecules such as stearoyl ethanolamide. This interaction could prevent sulfur metabolism from depleting already reduced levels of stearoyl ethanolomide and other lipids. A similar relationship between methionine metabolism and PEMT features in a putative relationship between hyperlipidemia and hyperhomocysteinemia [13] but has not previously been described in heat stress.

Changes to sulfur metabolism under heat stress can influence lipids in a number of ways beyond the SAH interaction with PEMT and are depicted in Fig 8. In the model created by the combined transcriptome and metabolome data, choline, the precursor to many fatty acids, is directed away from the production of signaling and structural lipids. This form of regulation is supported both by the behavior of the linear models and significant changes in gene expression.

For example, several shifts in gene expression can route this resource towards sulfur metabolism. Choline oxidase, the gene encoding the enzyme that oxidizes choline to produce betaine, is up-regulated (p-value = .018). Betaine plays an important role in the methionine cycle by providing an alternative pathway for methylation of homocysteine [17]. Concurrently, transcription of the first enzyme involved in converting choline to phosphatidylcholine, choline kinase, is down-regulated. Betaine levels, however, are unchanged (p-value = .815), suggesting redirected choline rescues betaine levels. Further supporting a relationship between sulfur and lipid metabolism via choline and betaine, Betaine—Homocysteine S-Methyltransferase (BHMT) transcription is downregulated under heat stress (p-value = .0163). BHMT converts betaine and homocysteine to dimethylglycine and methionine, and mouse knockouts of this gene show highly elevated levels of homocysteine [18].

Additionally, ethanolamine kinase (ETNK1) is up-regulated (p-value = .0182). This is consistent with gene expression changes preventing depletion of phosphatidylethanolamine-derived lipids such as phosphatidylcholine and stearoyl ethanolamide. Importantly, these models provide clarification and improvement over previous studies that have investigated the influence of amino acid and nutrient supplementation on heat stress performance. Such work has lacked mechanistic descriptions of how specific compounds relate to metabolism. For example, betaine and choline supplementation has been found to have variable effects on bird performance with recent studies suggesting it has limited influence on improving broiler performance and cannot overcome the negative influences of heat stress [19]. According to our extended model, the bird is able to effectively maintain betaine levels under heat stress through redirection of choline facilitated by gene expression changes. This creates a situation in which supplementation may be ineffective at further shifting network dynamics. We hypothesize gene regulation changes, as described through the transcriptome data, shunt choline to betaine, prevent the accumulation of resource deficits and provide a multi-omics explanation for the findings of [19]. These changes, previously described, include downregulation of BHMT, upregulation of choline oxidase and downregulation of choline kinase. The impact of these changes could be dramatic, given the proportion of lipid production that derives from these pathways. For example, modification of choline accounts for 70 percent of phospohatidylcholine synthesis, with the remaining 30 percent derived from PEMT driven methylation of phosphotidylethanolamine [20]. This latter pathway involves genes that demonstrate significant changes under heat stress, such as the upregulated choline kinase. These changes are consistent with adaptations to compensate for altered choline dynamics during long-term heat stress. Thus, the ability of choline, previously investigated, to rescue performance during stress may be stress and organism specific. For example, choline supplementation has been shown in clinical studies to improve antioxidant efficiency in cystic fibrosis patients [21] despite its efficacy in influencing livestock performance being equivocal [19].

### Advantages of multi-omics

Besides relating choline dynamics to sulfur metabolism, an important consequence of the regulatory circuit descrbed by multiple metabolic forks is selective processing of sulfur to increase the reservoir of anti-oxidants. This is most clearly evidenced by upregulation of glutathione (p-value = .00081). The coupling of this process to lipid metabolism is accomplished through changes in genes that sit at the intersection of the methionine cycle and choline metabolism. For example, as previously described, under heat stress, BHMT transcription is downregulated (p-value = .0176), potentially preserving cysteine pools while simultaneously managing the activity of the methionine to S-Adenosyl-L-methionine (SAM)/S-Adenosyl-L-homocysteine (SAH) cycle which could otherwise inhibit lipid producing Phosphatidylethanolamine N-methyltransferase (PEMT). The relationship between sulfur and lipid metabolism would not be evident without the combination of metabolite and transcriptome data. Importantly, the insights generated by this multi-omics work also propose mechanisms consistent with associations from GWAS (genome wide association studies). This is complementary to previous work on quantifying broiler performance under heat stress that has relied on QTL (quantitative trait loci) mapping to identify potentially important SNPs controlling relevant physiological metrics. One of these resides in the PEMT gene, which influences sulfur and lipid metabolism, as being associated with body temperature at Day 20 posthatch [22]. Our proposed circuit includes PEMT as a critical element in a broader network and provides a possible functional role of the previously identified SNP. Building circuits from individual network units provides biological context for statistical observations in a way that relate components from different, but connected, pathways. These network units can be further explored at the individual level to better understand the biological consequences of each model and their metabolic impact.

### Coordination of each fork as individual mechanisms

**Fig 9:**
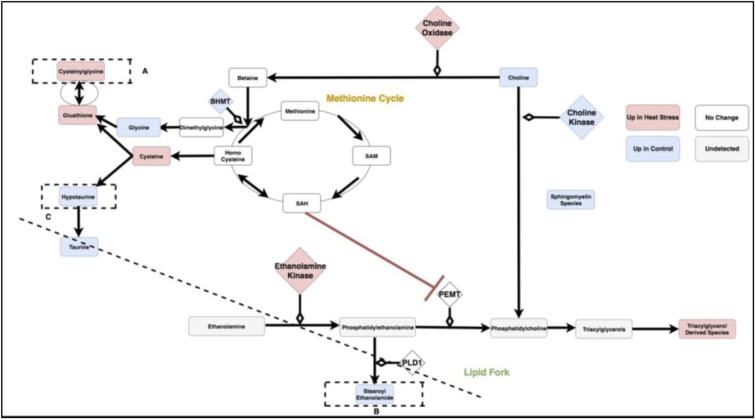
Triplet of cysteinylglycine and (stearoyl ethanolamide / hypotaurine). The compartmentalization of the pathway by regions containing the compounds in the ratio (stearoyl ethanolamide and hypotaurine) is illustrated by the dotted line. For the linear model representing differential behavior of this branch point, see Fig 4A. SAM: S-Adenosyl-L-methionine, SAH: S-Adenosyl-L-homocysteine,Glutathione GSSG: Glutathione Disulfide, PEMT: Phosphatidylethanolamine N-methyltransferase, BHMT: Betaine—Homocysteine S-Methyltransferase, PLD1: Phospholipase D-1, PEMT: Phosphatidylethanolamine N-methyltransferase.

Under heat stress conditions, stearoyl ethanolamide levels correlate well with ratios of the reduced glutathione derivative, cysteinylglcyine, and hypotaurine (Figs 9 and 6B). This latter quantity represents a metabolic fork underlying sulfur metabolism, which favors glutathione under heat stress. Under control conditions, activation of the sulfur metabolism would be predicted to inhibit an important component of stearoyl ethanolamide production via SAH-related inhibition of PEMT. This mechanism is countered under heat stress conditions with an increase in the ratio of PEMT/SAM correlating with rising levels of gamma glutamylcysteine (Fig 6F).

**Fig 10:**
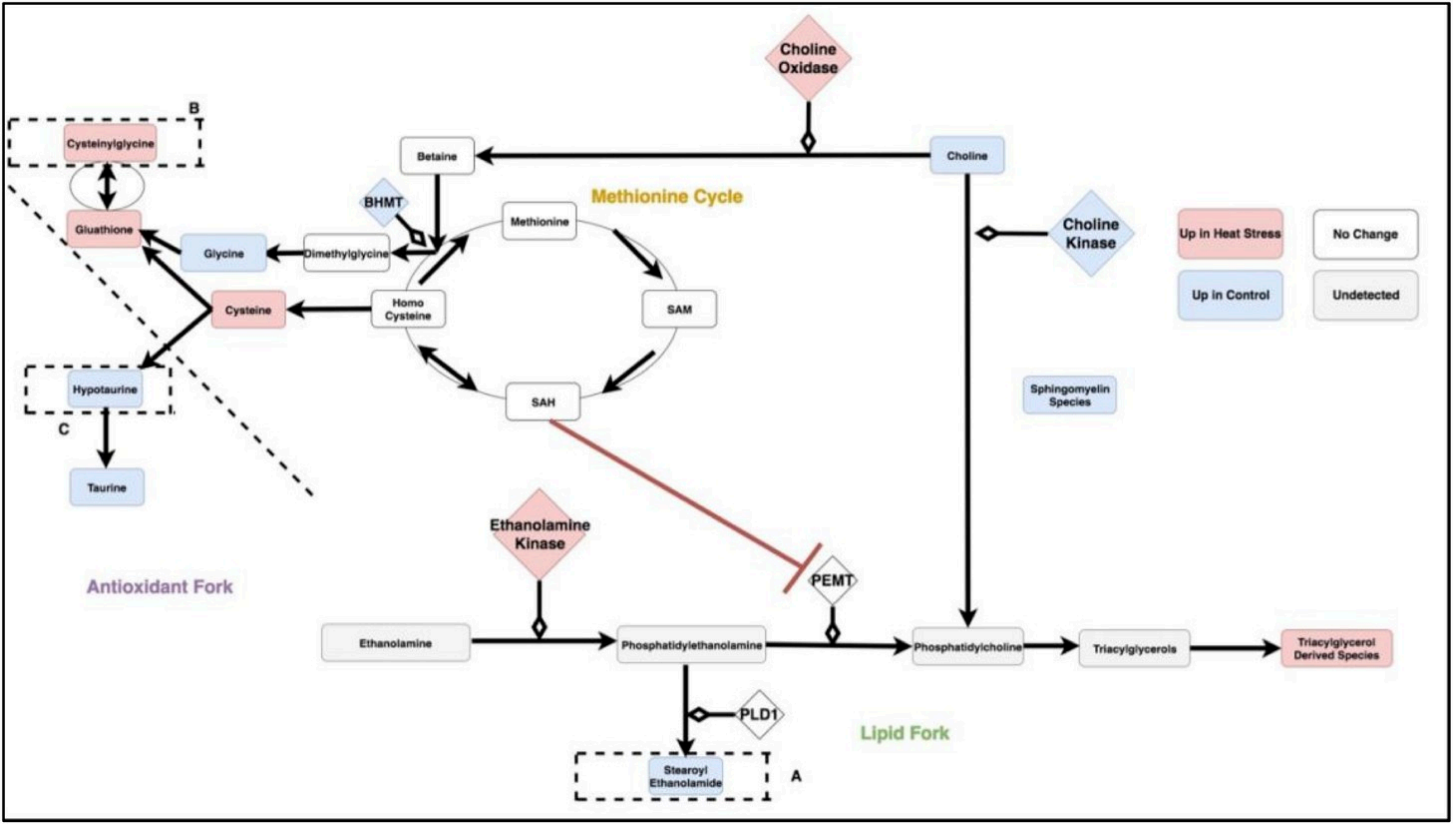
Triplet of stearoyl ethanolamide and (cysteinylglycine / hypotaurine). The compartmentalization of the pathway by regions containing the compounds in the ratio (cysteinylglycine and hypotaurine) is illustrated by the dotted line. For the linear model representing differential behavior of this branch point, see Fig 4B. SAM: S-Adenosyl-L-methionine, SAH: S-Adenosyl-L-homocysteine,Glutathione GSSG: Glutathione Disulfide, PEMT: Phosphatidylethanolamine N-methyltransferase, BHMT: Betaine—Homocysteine S-Methyltransferase, PLD1: Phospholipase D-1, PEMT: Phosphatidylethanolamine N-methyltransferase.

Stearoyl ethanolamide levels and the ratio of the reduced glutathione derivative, cysteinylglycine, and hypotaruine show strong patterns of differential correlation between control and heat stress (Figs 6C and 10). This is consistent with concerted regulation of several metabolic forks in the underlying circuit of carbon metabolism described below.

**Fig 11:**
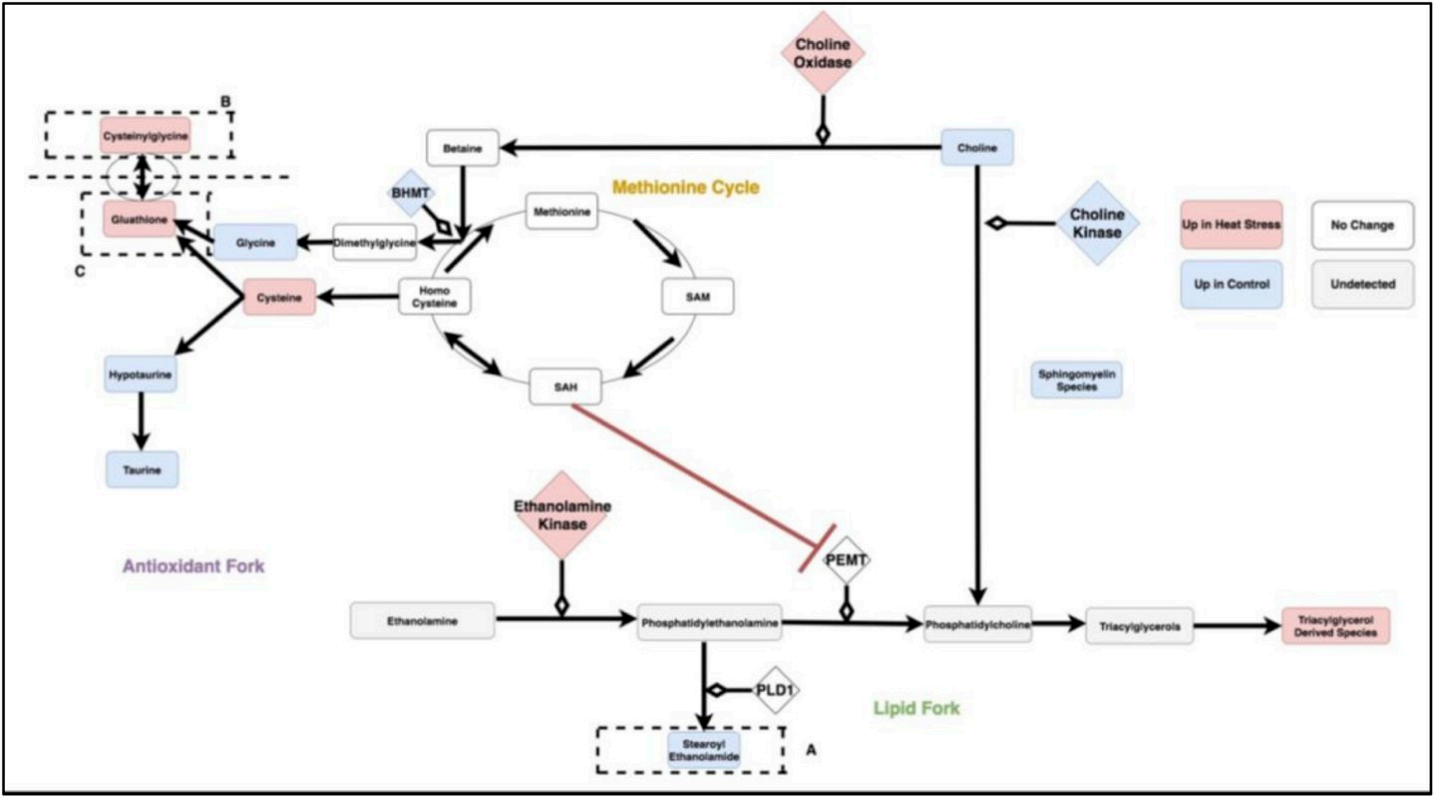
Triplet of stearoyl ethanolamide and (cysteinylglycine / gluathatione). The compartmentalization of the pathway by regions containing the compounds in the ratio (cysteinylglycine and glutathione) is illustrated by the dotted line. For the linear model representing differential behavior of this branch point, see Fig 4C. SAM: S-Adenosyl-L-methionine, SAH: S-Adenosyl-L-homocysteine,Glutathione GSSG: Glutathione Disulfide, PEMT: Phosphatidylethanolamine N-methyltransferase, BHMT: Betaine—Homocysteine S-Methyltransferase, PLD1: Phospholipase D-1, PEMT: Phosphatidylethanolamine N-methyltransferase.

Stearoyl ethanolamide levels and the ratio of the reduced glutathione derivative, cysteinylglycine, and Glutathione GSSG (Glutathione Disulfide) show strong patterns of differential correlation between control and heat stress (Figs 8 and 11). This is consistent with concerted regulation of several metabolic forks in the underlying circuit of carbon metabolism.

**Fig 12:**
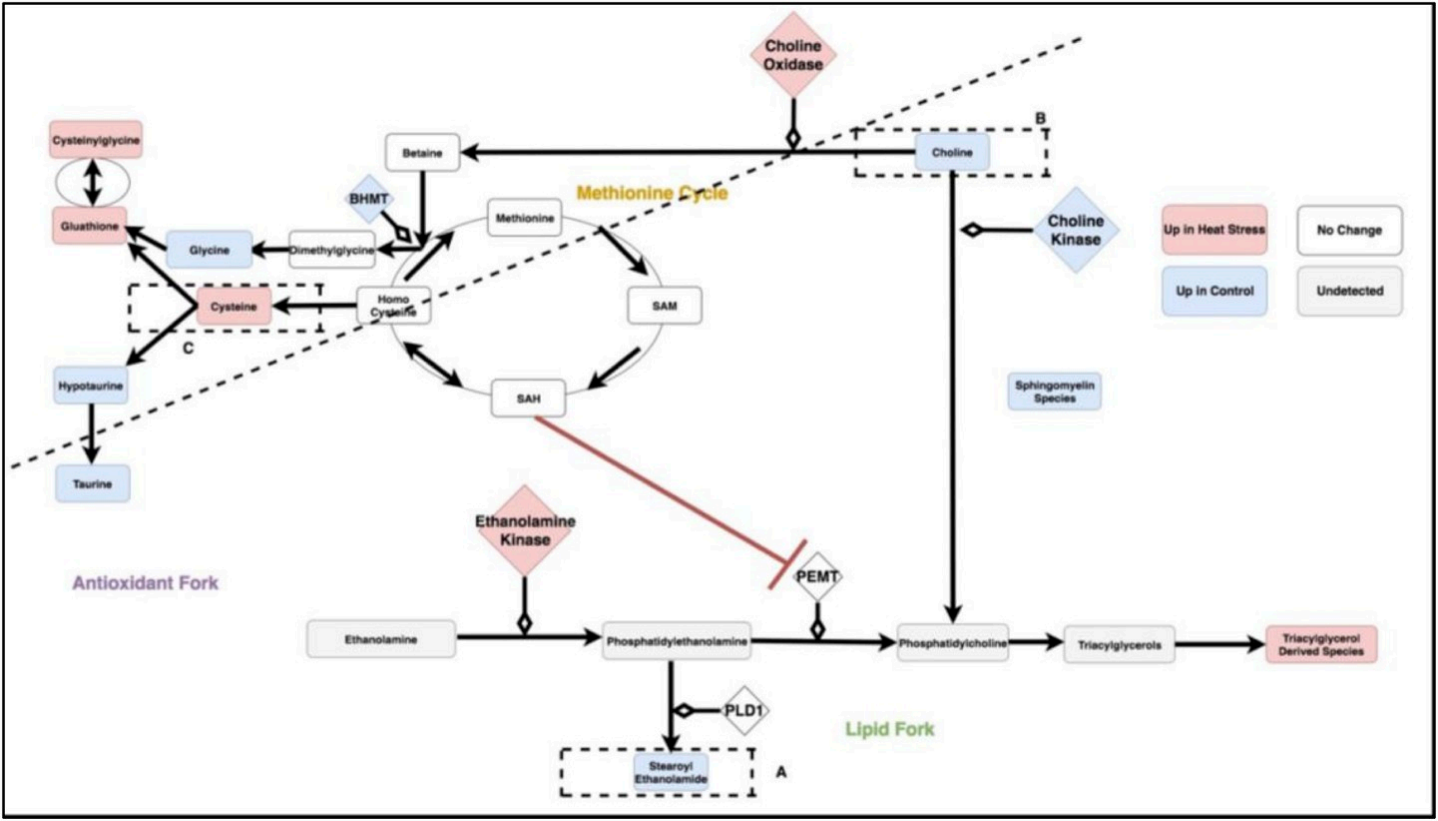
Triplet of stearoyl ethanolamide and (cysteine / choline). The compartmentalization of the pathway by regions containing the compounds in the ratio (choline and cysteine) is illustrated by the dotted line. For the linear model representing differential behavior of this branch point, see Fig 4D. SAM: S-Adenosyl-L-methionine, SAH: S-Adenosyl-L-homocysteine,Glutathione GSSG: Glutathione Disulfide, PEMT: Phosphatidylethanolamine N-methyltransferase, BHMT: Betaine—Homocysteine S-Methyltransferase, PLD1: Phospholipase D-1, PEMT: Phosphatidylethanolamine N-methyltransferase.

Stearoyl ethanolamide levels and the ratio of cysteine to choline shows strong patterns of differential correlation between control and heat stress (Figs 6D and 12). Under the proposed mechanism, as cysteine metabolism is increased during heat stress, choline availability decreases with its remaining levels leveraged to maintain betaine.

The coupling of stearoyl ethanolamide to sulfur metabolism is also consistent with the utilization of carbon backbones for gluconeogenesis and triacylglycerol production, as indicated by the upregulated phosphoethanolamine kinase (p-value = .0182). Under such a metabolic scheme, the bird allocates carbon resources to signaling molecules as well as to antioxidant and energy production pathways. Increased gluconeogenesis and lipogenesis patterns have been previously described in heat stressed broilers [9]. These findings are consistent with other studies that have implicated increased importance in sulfurous amino acid processing following down-regulation of fatty acid metabolism [23]. However, unlike previous studies we provide detailed descriptions of these mechanisms in the context of coordinated sulfur and lipid metabolism during chronic heat stress.

The circuit-oriented model of the heat stress response, derived by joining metabolic forks as network elements, provides a mechanistic context for correlations that would otherwise be enigmatic–such as the strong correlation between stearoyl ethanolamide and cysteine (Figs 6E and 7). Stearoyl ethanolamide and cysteine demonstrate differential relationships between control and heat stress conditions (p-value for the interaction term of the model steaoyl ethanolamide as a function of cysteine is .000917 and p-value for the interaction term in the model cysteine ~ stearoyl ethanolamide is .00203). Importantly, these relationships are now provided with mechanistic and physiological context that extends previous work to establish links between the complex features of stress management systems. For example, a central feature of the metabolite data of this study is that sulfur metabolism shifts towards glutathione and antioxidant production, but also has wide reaching consequences on other types compounds. Gluathathione has an established role in antioxidant activity of that plays a role in broiler physiology [24] and this study demonstrates how its regulation is related to lipid and general sulfur metabolism.

The central importance of antioxidants, at a molecular level, can be traced to production of reactive species, which are increasingly linked to the accelerated oxidation during heat stress [25]. These reactive species can result from changes in lipid metabolism, whereby oxidation of lipids becomes an important mitochondrial event during heat stress [26]. The coupling of sulfur and lipid metabolism in this dataset is consistent with this earlier work, but provides a more thorough description of their regulation.

The physiological roles of stearoyl ethanolamide are not fully established, although it has been shown to have anti-inflammatory properties [27]. By describing its association with heat stress responsive pathways, we provide pathway specific context for its production, and other lipids in general (such as choline and sphingomyelins). Our computational analysis provides insight into changes that may influence physiology under heat stress. This analysis provides network context for previous studies that have identified differential gene expression and metabolite levels [9]. Future work will be directed towards modeling the influence of these metabolite and gene expression changes on bird physiology through blood serum measurements.

## Conclusions

Systems biology studies can use multi-omics data to identify elements of regulation, integrating them into concrete networks that generate hypotheses about large-scale regulation. Collectively, these changes in gene expression and metabolic forks identified by this work provide mechanistic context for the differential relationship between stearoyl ethanolamide and cysteine during heat stress. The insights from this study expand the role of carbon of and sulfur flux during the long-term heat stress response.

Leveraging computational methods to understand the nuances of carbon and sulfur flow under heat stress provides a significant improvement in understanding the regulation of the response, and generates a number of testable hypotheses. These are being incorporated to plan studies in which feed composition is altered with resources thought to be involved in the major circuits. Additionally, we have successfully captured the logic of the carbon flow under heat stress. The transition from simply determining up or down regulation of certain compounds developing a collection of well-characterized mechanisms to be integrated into circuits is a powerful improvement in using systems biology to integrate large- scale multi-omics data.

## Additional materials and methods

### Ethics statement

This study was carried out in strict accordance with the recommendations in the Guide for the Care and Use of Laboratory Animals of the National Institutes of Health. The protocol was approved by the Committee on the Ethics of Animal Experiments of the University of Delaware (Permit Number: 2703-12-10).

### Bird and tissue handling

Male broiler chickens *(Gallus gallus)* were obtained from Mountaire hatchery (Millsboro, DE) on day of hatch and divided into thermoneutral and experimental houses on the University of Delaware farm. They were raised under a light cycle of 23 hours of light and 1 hour of dark. Standard management and husbandry procedures were followed, as approved by the Animal Care and Use Committee (AACUC #(27) 03-12-14R). Birds were given *ad libitum* access to water and fed the same diet (corn-soy) which met all NRC requirements [28]. Both groups were raised at 35°C until one-week post hatch. Temperature was decreased 5°C each week thereafter until temperature reached 25°C at day 21 post hatch. The thermoneutral house was then maintained at 25°C and the heat stress house was subject to 35–37°C for 8 hours per day, to mimic an environmental heat wave. Temperature in both houses was maintained by a computerized system controlling heaters and ventilation fans (Chore-time Equipment, Milford, Indiana). Temperature ranged between 35–37°C during the eight hours of heat stress. This yields an internal body temperature (cloacal) of 43.5°C within two hours of the onset of heat stress. This body temperature can induce a heat stress response in chicken cells [29]. In the control (thermoneutral) house the temperature ranged between 23–25°C during this same period. Both houses were maintained at 23–25°C during the thermoneutral period (16 hours) of the day. Birds were euthanized via cervical dislocation and necropsied at day 28 post hatch, following one week of cyclic heat stress. Livers were flash frozen in liquid nitrogen, and stored at -80°C for further processing.

### RNA and library preparation

Forty-five mg of the left lobe of 8 thermoneutral and 8 heat stress liver samples were homogenized and RNA was extracted using the mirVana miRNA Isolation Kit (Ambion, Austin, TX) as per manufacturer instructions. They were quantified using the Qubit 2.0 Fluorometer (Qubit, New York, NY). Samples were checked for quality using the Fragment Analyzer (Advanced Analytical, Ankeny, IA) at the Delaware Biotechnology Institute (DBI, Newark, DE). Libraries were made using the Illumina TruSeq Stranded mRNA Sample Preparation Kit (Illumina, San Diego, CA) per manufacturer instructions and sent to DBI for sequencing.

### Metabolome sample preparation

Fifty mg of 12 thermoneutral and 11 heat stress liver samples were sent to Metabolon (Durham, NC), for analysis of the metabolome. All of the samples used for the transcriptome analysis were included in the metabolomic sample set. Samples were analyzed as previously described [30]. Samples were prepared using the MicroLab STAR system from Hamilton Company (Reno, NV) using in house recovery standards prior to extraction for QC purposes. Extract was divided into fractions for two reverse phase (RP)/UPLC-MS/MS methods (positive and negative ion mode electrospray ionization), and one for HILIC/UPLC-MS/MS with negative ion mode ESI. Several controls were used, including the use of technical replicates, extracted water samples as blanks, and in house QC samples to monitor chromatographic alignment. All UPLC-MS/MS methods used a waters ACQUITY UPLC and Thermo Scientific Q-Exactive high-resolution mass spectrometer. Each sample extract was dried and reconstituted with solvents compatible to each method and solvents included a series of standards at fixed concentrations. Metabolon used hardware and software extract created by the company to extract, peak-identify, and QC process the raw data. Compounds were identified using a Metabolon maintained library of purified standards or recurrent unknown entries. Data is provided as a supplementary .txt file. Over 3300 compounds have been identified and registered in Metabolon’s library. The data was statistically analyzed using a Welch’s two-sample t-test following a log transformation and imputation of missing values with the minimum observed value for each compound. The company provided an analysis that included pathway visualizations. These pathway analyses were then incorporated with the transcriptome data to create a more complete view of changing pathways.

### Transcriptome analysis

Once libraries were sequenced, data were processed using an in-house pipeline and fragments per kilobase per million mapped reads (FPKM) values were determined. Differential expression was determined by using the standard t.test() function in R. Correlation values for triplets of the form $cor\;\left(A,(\frac{B}{C})\right)$, where A,B and C represents the levels of metabolites or gene transcripts were also assessed in R.

## Supporting information

S1 FigModel 1A.Model information for model of the form $A∼\left(\frac{B}{C}\right)$, where A = stearoyl ETOH, B = cysteinylglycine, C = hypotaurine.(PDF)

S2 FigModel 1B.Model information for model of the form $\left(\frac{B}{C}\right)∼A$, where A = stearoyl ETOH, B = cysteinylglycine, C = hypotaurine.(PDF)

S3 FigModel 2A.Model information for model of the form $A∼\left(\frac{B}{C}\right)$, where stearoyl EtOH, B = glutathione GSSG, C = cysteinylglycine.(PDF)

S4 FigModel 2B.Model information for model of the form $\left(\frac{B}{C}\right)∼A$, where stearoyl EtOH, B = glutathione GSSG, C = cysteinylglycine.(PDF)

S5 FigModel 3A.Model information for model of the form $A∼\left(\frac{B}{C}\right)$, where A = stearoyl ethanolamide, B = cysteine, C = choline.(PDF)

S6 FigModel 3B.Model information for model of the form $\left(\frac{B}{C}\right)∼A$, where A = stearoyl ethanolamide, B = cysteine, C = choline.(PDF)

S7 FigModel 4A.Model information for model of the form $A∼\left(\frac{B}{C}\right)$, where A = stearoyl ethoh, B = cysteinylglycine and C = taurine.(PDF)

S8 FigModel 4B.Model information for model of the form $\left(\frac{B}{C}\right)∼A$, where A = stearoyl ethoh, B = cysteinylglycine and C = taurine.(PDF)

S9 Fig Model 5A. Model information for model of the form A ~ B, where A = stearoyl ethoh, B = cysteine.
(PDF)

S10 Fig Model 5B. Model information for model of the form B ~ A, where A = stearoyl ethoh, B = cysteine.
(PDF)

S11 FigModel 6A.Model information for model of the form $A∼\left(\frac{B}{C}\right)$, where A = cysteinylglycine B = PEMT and C = SAM.(PDF)

S12 FigModel 6B.Model information for model of the form $\left(\frac{B}{C}\right)∼A$, where A = cysteinylglycine B = PEMT and C = SAM.(PDF)

S1 Table Metabolite data. (CSV)

S2 Table Gene expression data. (TXT)

## Abbreviations

ChO: Choline Oxidase
ChoK: Choline Kinase
SNP: Single Nucleotide Polymorphism
Glutathione GSSG: Glutathione Disulfide
SAH: S-Adenosyl-L-homocysteine
SAM: S-Adenosyl-L-methionine
PEMT: Phosphatidylethanolamine N-methyltransferase
ETNK1: Ethanolamine Kinase
BHMT: Betaine—Homocysteine S-Methyltransferase
PLD-1: Phospholipase-D1
GWAS: Genome Wide Association Studies
QTL: Quantitative Trait Loci
