# Supplementary material for: Identifying mechanisms of regulation to model carbon flux during heat stress and generate testable hypotheses
Source: PLoS One. 2018 Oct 26;13(10):e0205824. doi: 10.1371/journal.pone.0205824 (PMC6203350; doi:10.1371/journal.pone.0205824)
Supplement: S1 Fig — Model information for model of the form A∼(BC), where A = stearoyl ETOH, B = cysteinylglycine, C = hypotaurine. (PDF) [file pone.0205824.s001.pdf]

```
Call:
lm(formula = A ~ BDivC * theIndicator, data = theSubset)

Residuals:
     Min       1Q   Median       3Q      Max 
-0.11718 -0.07446 -0.03671  0.04725  0.24701 

Coefficients:
                    Estimate Std. Error t value Pr(>|t|)    
(Intercept)          13.7593     0.1879  73.243   <2e-16 ***
BDivC                 0.2843     0.1144   2.484   0.0287 *  
theIndicator1        -0.4099     0.3175  -1.291   0.2210    
BDivC:theIndicator1  -0.3910     0.1427  -2.741   0.0179 *  
---
Signif. codes:  0 ‘***’ 0.001 ‘**’ 0.01 ‘*’ 0.05 ‘.’ 0.1 ‘ ’ 1

Residual standard error: 0.1166 on 12 degrees of freedom
Multiple R-squared:  0.794,	Adjusted R-squared:  0.7425 
F-statistic: 15.42 on 3 and 12 DF,  p-value: 0.0002032
```
