# Supplementary material for: Identifying mechanisms of regulation to model carbon flux during heat stress and generate testable hypotheses
Source: PLoS One. 2018 Oct 26;13(10):e0205824. doi: 10.1371/journal.pone.0205824 (PMC6203350; doi:10.1371/journal.pone.0205824)
Supplement: S2 Fig — Model information for model of the form (BC)∼A, where A = stearoyl ETOH, B = cysteinylglycine, C = hypotaurine. (PDF) [file pone.0205824.s002.pdf]

```
Call:
lm(formula = BDivC ~ theIndicator * A, data = theSubset)

Residuals:
     Min       1Q   Median       3Q      Max 
-0.81871 -0.16208  0.06757  0.16056  0.63758 

Coefficients:
                Estimate Std. Error t value Pr(>|t|)  
(Intercept)      -31.539     14.834  -2.126   0.0549 .
theIndicator1     48.488     21.024   2.306   0.0397 *
A                  2.250      1.115   2.018   0.0665 .
theIndicator1:A   -3.707      1.559  -2.378   0.0349 *
---
Signif. codes:  0 ‘***’ 0.001 ‘**’ 0.01 ‘*’ 0.05 ‘.’ 0.1 ‘ ’ 1

Residual standard error: 0.4038 on 12 degrees of freedom
Multiple R-squared:  0.811,     Adjusted R-squared:  0.7637 
F-statistic: 17.16 on 3 and 12 DF,  p-value: 0.0001225
```
