# Supplementary material for: Identifying mechanisms of regulation to model carbon flux during heat stress and generate testable hypotheses
Source: PLoS One. 2018 Oct 26;13(10):e0205824. doi: 10.1371/journal.pone.0205824 (PMC6203350; doi:10.1371/journal.pone.0205824)
Supplement: S3 Fig — Model information for model of the form A∼(BC), where stearoyl EtOH, B = glutathione GSSG, C = cysteinylglycine. (PDF) [file pone.0205824.s003.pdf]

```
Call:
lm(formula = A ~ BDivC * theIndicator, data = theSubset)

Residuals:
     Min       1Q   Median       3Q      Max 
-0.12992 -0.06838 -0.01966  0.05211  0.23555 

Coefficients:
                    Estimate Std. Error t value Pr(>|t|)    
(Intercept)          14.4315     0.3832  37.664 7.89e-14 ***
BDivC                -0.3952     0.1337  -2.957  0.01199 *  
theIndicator1        -1.2349     0.4748  -2.601  0.02319 *  
BDivC:theIndicator1   0.5163     0.1517   3.404  0.00523 ** 
---
Signif. codes:  0 ‘***’ 0.001 ‘**’ 0.01 ‘*’ 0.05 ‘.’ 0.1 ‘ ’ 1

Residual standard error: 0.1067 on 12 degrees of freedom
Multiple R-squared:  0.8277,	Adjusted R-squared:  0.7846 
F-statistic: 19.21 on 3 and 12 DF,  p-value: 7.091e-05
```
