# Supplementary material for: Identifying mechanisms of regulation to model carbon flux during heat stress and generate testable hypotheses
Source: PLoS One. 2018 Oct 26;13(10):e0205824. doi: 10.1371/journal.pone.0205824 (PMC6203350; doi:10.1371/journal.pone.0205824)
Supplement: S4 Fig — Model information for model of the form (BC)∼A, where stearoyl EtOH, B = glutathione GSSG, C = cysteinylglycine. (PDF) [file pone.0205824.s004.pdf]

```
Call:
lm(formula = BDivC ~ theIndicator * A, data = theSubset)

Residuals:
     Min       1Q   Median       3Q      Max 
-0.53916 -0.17809 -0.02324  0.15054  0.92634 

Coefficients:
                Estimate Std. Error t value Pr(>|t|)  
(Intercept)       28.377     14.403   1.970   0.0723 .
theIndicator1    -51.186     20.412  -2.508   0.0275 *
A                 -1.919      1.083  -1.772   0.1017  
theIndicator1:A    3.871      1.514   2.557   0.0251 *
---
Signif. codes:  0 ‘***’ 0.001 ‘**’ 0.01 ‘*’ 0.05 ‘.’ 0.1 ‘ ’ 1

Residual standard error: 0.3921 on 12 degrees of freedom
Multiple R-squared:  0.7381,	Adjusted R-squared:  0.6726 
F-statistic: 11.27 on 3 and 12 DF,  p-value: 0.0008339
```
