# Supplementary material for: Identifying mechanisms of regulation to model carbon flux during heat stress and generate testable hypotheses
Source: PLoS One. 2018 Oct 26;13(10):e0205824. doi: 10.1371/journal.pone.0205824 (PMC6203350; doi:10.1371/journal.pone.0205824)
Supplement: S5 Fig — Model information for model of the form A∼(BC), where A = stearoyl ethanolamide, B = cysteine, C = choline. (PDF) [file pone.0205824.s005.pdf]

```
Call:
lm(formula = A ~ BDivC * theIndicator, data = theSubset)

Residuals:
     Min       1Q   Median       3Q      Max 
-0.15534 -0.08057  0.01133  0.05003  0.17165 

Coefficients:
                    Estimate Std. Error t value Pr(>|t|)    
(Intercept)          14.0039     0.2376  58.933 3.77e-16 ***
BDivC                 0.4311     0.1447   2.979  0.01151 *  
theIndicator1        -0.8600     0.3263  -2.635  0.02175 *  
BDivC:theIndicator1  -0.6602     0.1742  -3.791  0.00257 ** 
---
Signif. codes:  0 ‘***’ 0.001 ‘**’ 0.01 ‘*’ 0.05 ‘.’ 0.1 ‘ ’ 1

Residual standard error: 0.1007 on 12 degrees of freedom
Multiple R-squared:  0.8463,	Adjusted R-squared:  0.8079 
F-statistic: 22.03 on 3 and 12 DF,  p-value: 3.596e-05
```
