# Supplementary material for: Identifying mechanisms of regulation to model carbon flux during heat stress and generate testable hypotheses
Source: PLoS One. 2018 Oct 26;13(10):e0205824. doi: 10.1371/journal.pone.0205824 (PMC6203350; doi:10.1371/journal.pone.0205824)
Supplement: S6 Fig — Model information for model of the form (BC)∼A, where A = stearoyl ethanolamide, B = cysteine, C = choline. (PDF) [file pone.0205824.s006.pdf]

```
Call:
lm(formula = BDivC ~ A * theIndicator, data = theSubset)

Residuals:
     Min       1Q   Median       3Q      Max 
-0.45697 -0.13364  0.08784  0.13023  0.37016 

Coefficients:
                Estimate Std. Error t value Pr(>|t|)   
(Intercept)     -22.7969     9.4025  -2.425  0.03205 * 
A                 1.5915     0.7067   2.252  0.04384 * 
theIndicator1    45.1630    13.3254   3.389  0.00538 **
A:theIndicator1  -3.3949     0.9883  -3.435  0.00494 **
---
Signif. codes:  0 ‘***’ 0.001 ‘**’ 0.01 ‘*’ 0.05 ‘.’ 0.1 ‘ ’ 1

Residual standard error: 0.2559 on 12 degrees of freedom
Multiple R-squared:  0.7605,	Adjusted R-squared:  0.7007 
F-statistic:  12.7 on 3 and 12 DF,  p-value: 0.0004929
```
