# Supplementary material for: Identifying mechanisms of regulation to model carbon flux during heat stress and generate testable hypotheses
Source: PLoS One. 2018 Oct 26;13(10):e0205824. doi: 10.1371/journal.pone.0205824 (PMC6203350; doi:10.1371/journal.pone.0205824)
Supplement: S7 Fig — Model information for model of the form A∼(BC), where A = stearoyl ethoh, B = cysteinylglycine and C = taurine. (PDF) [file pone.0205824.s007.pdf]

```
Call:
lm(formula = A ~ BDivC * theIndicator, data = theSubset)

Residuals:
     Min       1Q   Median       3Q      Max 
-0.20583 -0.08055 -0.01577  0.07602  0.27675 

Coefficients:
                    Estimate Std. Error t value Pr(>|t|)    
(Intercept)         13.95025    0.36586  38.130 6.81e-14 ***
BDivC               -0.14857    0.08346  -1.780   0.1004    
theIndicator1       -0.70814    0.55437  -1.277   0.2256    
BDivC:theIndicator1  0.22008    0.10885   2.022   0.0661 .  
---
Signif. codes:  0 ‘***’ 0.001 ‘**’ 0.01 ‘*’ 0.05 ‘.’ 0.1 ‘ ’ 1

Residual standard error: 0.1286 on 12 degrees of freedom
Multiple R-squared:  0.7493,	Adjusted R-squared:  0.6866 
F-statistic: 11.95 on 3 and 12 DF,  p-value: 0.0006456
```
