# Supplementary material for: Identifying mechanisms of regulation to model carbon flux during heat stress and generate testable hypotheses
Source: PLoS One. 2018 Oct 26;13(10):e0205824. doi: 10.1371/journal.pone.0205824 (PMC6203350; doi:10.1371/journal.pone.0205824)
Supplement: S8 Fig — Model information for model of the form (BC)∼A, where A = stearoyl ethoh, B = cysteinylglycine and C = taurine. (PDF) [file pone.0205824.s008.pdf]

```
Call:
lm(formula = BDivC ~ theIndicator * A, data = theSubset)

Residuals:
    Min      1Q  Median      3Q     Max 
-0.7199 -0.3301 -0.1828  0.2371  1.3751 

Coefficients:
                Estimate Std. Error t value Pr(>|t|)  
(Intercept)       40.156     22.214   1.808   0.0958 .
theIndicator1    -58.374     31.482  -1.854   0.0884 .
A                 -2.691      1.670  -1.612   0.1329  
theIndicator1:A    4.458      2.335   1.909   0.0804 .
---
Signif. codes:  0 ‘***’ 0.001 ‘**’ 0.01 ‘*’ 0.05 ‘.’ 0.1 ‘ ’ 1

Residual standard error: 0.6047 on 12 degrees of freedom
Multiple R-squared:  0.7205,	Adjusted R-squared:  0.6506 
F-statistic: 10.31 on 3 and 12 DF,  p-value: 0.00122
```
