# Supplementary material for: Identifying mechanisms of regulation to model carbon flux during heat stress and generate testable hypotheses
Source: PLoS One. 2018 Oct 26;13(10):e0205824. doi: 10.1371/journal.pone.0205824 (PMC6203350; doi:10.1371/journal.pone.0205824)
Supplement: S9 Fig — Model information for model of the form A ~ B, where A = stearoyl ethoh, B = cysteine. (PDF) [file pone.0205824.s009.pdf]

```
Call:
lm(formula = A ~ B * theIndicator, data = theSubset)

Residuals:
     Min       1Q   Median       3Q      Max 
-0.15798 -0.01515  0.01436  0.03900  0.18003 

Coefficients:
                Estimate Std. Error t value Pr(>|t|)    
(Intercept)       2.8948     3.0631   0.945 0.363272    
B                 0.5632     0.1657   3.398 0.005285 ** 
theIndicator1    16.2078     3.6324   4.462 0.000776 ***
B:theIndicator1  -0.8632     0.1977  -4.367 0.000917 ***
---
Signif. codes:  0 ‘***’ 0.001 ‘**’ 0.01 ‘*’ 0.05 ‘.’ 0.1 ‘ ’ 1

Residual standard error: 0.09259 on 12 degrees of freedom
Multiple R-squared:  0.8701,    Adjusted R-squared:  0.8376 
F-statistic:  26.8 on 3 and 12 DF,  p-value: 1.327e-05
```
