# Supplementary material for: Identifying mechanisms of regulation to model carbon flux during heat stress and generate testable hypotheses
Source: PLoS One. 2018 Oct 26;13(10):e0205824. doi: 10.1371/journal.pone.0205824 (PMC6203350; doi:10.1371/journal.pone.0205824)
Supplement: S10 Fig — Model information for model of the form B ~ A, where A = stearoyl ethoh, B = cysteine. (PDF) [file pone.0205824.s010.pdf]

```
Call:
lm(formula = B ~ A * theIndicator, data = theSubset)

Residuals:
     Min       1Q   Median       3Q      Max 
-0.41827 -0.05564 -0.00123  0.08347  0.27257 

Coefficients:
                Estimate Std. Error t value Pr(>|t|)   
(Intercept)       0.6505     7.1722   0.091  0.92922   
A                 1.3403     0.5391   2.486  0.02863 * 
theIndicator1    39.5513    10.1646   3.891  0.00214 **
A:theIndicator1  -2.9558     0.7539  -3.921  0.00203 **
---
Signif. codes:  0 ‘***’ 0.001 ‘**’ 0.01 ‘*’ 0.05 ‘.’ 0.1 ‘ ’ 1

Residual standard error: 0.1952 on 12 degrees of freedom
Multiple R-squared:  0.7073,	Adjusted R-squared:  0.6341 
F-statistic: 9.665 on 3 and 12 DF,  p-value: 0.001596
```
