# Supplementary material for: Identifying mechanisms of regulation to model carbon flux during heat stress and generate testable hypotheses
Source: PLoS One. 2018 Oct 26;13(10):e0205824. doi: 10.1371/journal.pone.0205824 (PMC6203350; doi:10.1371/journal.pone.0205824)
Supplement: S11 Fig — Model information for model of the form A∼(BC), where A = cysteinylglycine B = PEMT and C = SAM. (PDF) [file pone.0205824.s011.pdf]

```
Call:
lm(formula = A ~ BDivC * theIndicator, data = theSubset)

Residuals:
      Min        1Q    Median        3Q       Max 
-0.026146 -0.015226 -0.002512  0.011886  0.039076 

Coefficients:
                    Estimate Std. Error t value Pr(>|t|)    
(Intercept)          2.88744    0.14433  20.006 1.39e-10 ***
BDivC                0.01808    0.01460   1.238 0.239383    
theIndicator1       -0.97329    0.19375  -5.024 0.000297 ***
BDivC:theIndicator1 -0.09288    0.01970  -4.716 0.000501 ***
---
Signif. codes:  0 ‘***’ 0.001 ‘**’ 0.01 ‘*’ 0.05 ‘.’ 0.1 ‘ ’ 1

Residual standard error: 0.01999 on 12 degrees of freedom
Multiple R-squared:  0.8621,	Adjusted R-squared:  0.8276 
F-statistic: 25.01 on 3 and 12 DF,  p-value: 1.893e-05
```
