# Supplementary material for: Identifying mechanisms of regulation to model carbon flux during heat stress and generate testable hypotheses
Source: PLoS One. 2018 Oct 26;13(10):e0205824. doi: 10.1371/journal.pone.0205824 (PMC6203350; doi:10.1371/journal.pone.0205824)
Supplement: S12 Fig — Model information for model of the form (BC)∼A, where A = cysteinylglycine B = PEMT and C = SAM. (PDF) [file pone.0205824.s012.pdf]

```
Call:
lm(formula = BDivC ~ theIndicator * A, data = theSubset)

Residuals:
     Min       1Q   Median       3Q      Max 
-0.50551 -0.26503 -0.05135  0.20155  0.83465 

Coefficients:
                Estimate Std. Error t value Pr(>|t|)  
(Intercept)      -42.748     20.137  -2.123   0.0553 .
theIndicator1     62.337     21.806   2.859   0.0144 *
A                 12.137      7.433   1.633   0.1285  
theIndicator1:A  -23.236      8.078  -2.876   0.0139 *
---
Signif. codes:  0 ‘***’ 0.001 ‘**’ 0.01 ‘*’ 0.05 ‘.’ 0.1 ‘ ’ 1

Residual standard error: 0.3928 on 12 degrees of freedom
Multiple R-squared:   0.56,     Adjusted R-squared:   0.45 
F-statistic:  5.09 on 3 and 12 DF,  p-value: 0.01679
```
